# Plantar plate radiofrequency and Weil osteotomy for subtle metatarsophalangeal joint instablity

**DOI:** 10.1186/s13018-015-0318-1

**Published:** 2015-11-19

**Authors:** Caio Nery, Fernando C. Raduan, Fernanda Catena, Tania Szejnfeld Mann, Marco Antonio Percope de Andrade, Daniel Baumfeld

**Affiliations:** UNIFESP–Escola Paulista de Medicina, São Paulo, SP Brazil; UFMG–Federal University of Minas Gerais, Juvenal dos Santos St, 325, Belo Horizonte, MG 30380 5030 Brazil

**Keywords:** Joint instability, Metatarsophalangeal joint, Arthroscopy

## Abstract

**Background:**

To the present day, literature has only discussed how to treat extensive plantar plate and collateral ligament lesions, with gross joint subluxation and obvious clinical instability. The treatment options for early stages of the disease with minor injuries and subtle instabilities have not been described. The main purpose of this prospective study is to evaluate the efficacy of the combination of the arthroscopic radiofrequency shrinkage and distal Weil osteotomy in the treatment of subtle metatarsophalangeal joint instability.

**Method:**

Prospective data (clinical, radiological, and arthroscopic findings) of 19 patients, with a total of 35 slightly unstable joints, was collected. The physical examination defined the hypothesis for plantar plate lesions (grades 0 and 1), which was confirmed during the diagnostic step of the arthroscopic procedure.

**Results:**

Among our patients, 73 % were females and 63 % reported wearing high heels. The average age was 59 years and post-operative follow-up was 20 months. In the initial sample frame, 62 % of joints showed spread-out toes with increased interdigital spacing. The mean American Orthopedic Foot and Ankle Society score rose from 53 points pre-operatively to 92 points post-operatively and a visual-analog pain scale average value of eight points pre-operatively decreased to zero post-operatively. During the pre-operative evaluation, none of the patients had stable joints and over 97 % were classified as having grade 1 instability (<50 % subluxation). After treatment, 83 % of the joints became stable (degree of instability 0) and over 97 % were congruent. All studied parameters showed statistically significant improvements in the post-operative period (*p* < 0.001) showing the efficiency of the treatment in pain relief, while restoring the joint stability and congruity.

**Conclusion:**

Arthroscopic radiofrequency shrinkage in combination with distal Weil osteotomy promotes functional improvement, pain relief, and restores the joint stability in the plantar plate lesion grades 0 and 1.

## Introduction

In the last decade, we observed a crescent interest in the lesser toe deformities, particularly metatarsophalangeal (MTP) joint instability [1–5]. Recently, the involvement and importance of the plantar plate (PP) in lesser MTP joint instability has been evaluated and classified [6]. In the same way, grading schemes describing MTP joint instability have been reported [7, 8]. The main purposes of these grading systems are to address the pathophysiology of the lesions and to stratify different stages of soft tissue involvement, in order to try and create a treatment algorithm with alternatives for each different stage of MTP plantar plate lesion.

Until now, there has been no current treatment available in the literature to address the initial instability of the MTP joint. All reports describe the treatment for more extensive lesions with a clear clinical presentation without stratifying the grade of joint involvement [3, 8–10]. For the early stages of lesser MTP joint instability, conservative management is recommended with toe taping or corticoid injection, but these methods cannot prevent deformity progression [6, 11].

Electro-thermal capsular shrinkage (ECS) has become a common procedure for a variety of joint conditions usually associated with instability [12]. It is well known that radiofrequency in supra-physiological temperatures results in active wound healing by triggering the cellular response [13–17]. Based on these findings, we hypothesized that radiofrequency could affect the injured plantar plate in the early stages of MTP instability, preventing the progression of deformity.

The aim of this prospective study was to show the results obtained in the treatment of a group of patients with subtle plantar plate pathology with the arthroscopic radiofrequency shrinkage and sealing of the lesions combined with Weil metatarsal osteotomy.

## Methods

### Patient population

From January 2009 to June 2011, we prospectively enrolled 19 patients (with a total of 35 MTP joints) with subtle lesser metatarsophalangeal joint instability, after IRB approval of our institution (CAAE−18191613.0.0000.5149). All patients had forefoot pain and different degrees of MTP joint deformity and instability.

During patient interview, we analyzed the length of symptoms and the location of pain. A 10-point visual-analog pain scale (VAS) was used to grade the magnitude of pain and the AOFAS forefoot score was used to evaluate pre- and post-operative clinical results.

**Fig. 1:**
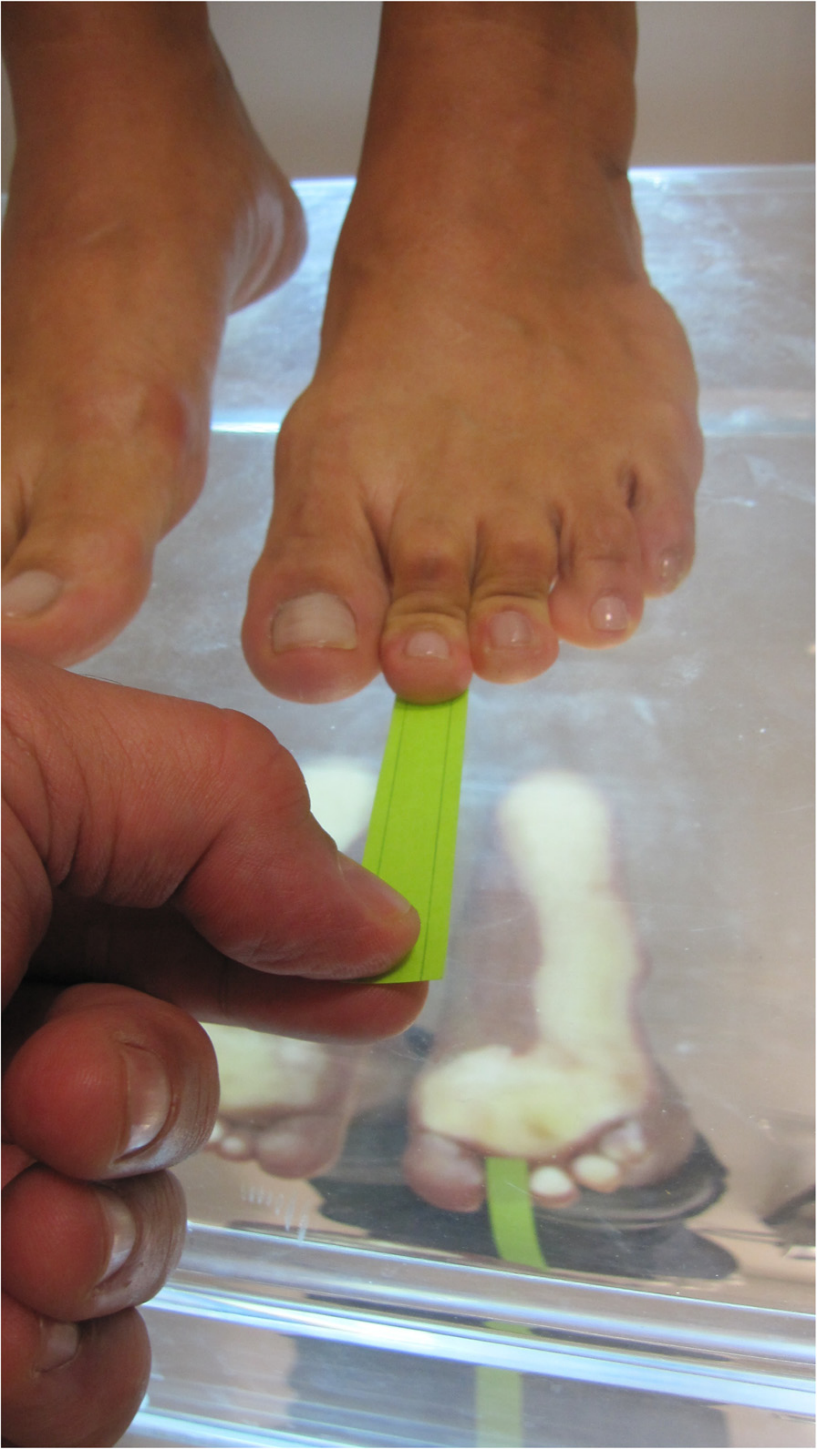
Toe-purchase: the strength of toe or digital purchase was evaluated using the “paper pullout test”

During the physical examination (both pre- and post-operatively), we assessed the presence of lesser toe misalignments in different planes: axial (varus/valgus), frontal (supination/pronation), and sagittal (dorsal/plantar) plane; the ability of the toe to touch the ground and the strength of the toe purchase and joint instability. Toe purchase was evaluated as described by Bouche and Heit [18] using the “paper pullout test” (Fig. 1).

**Fig. 2:**
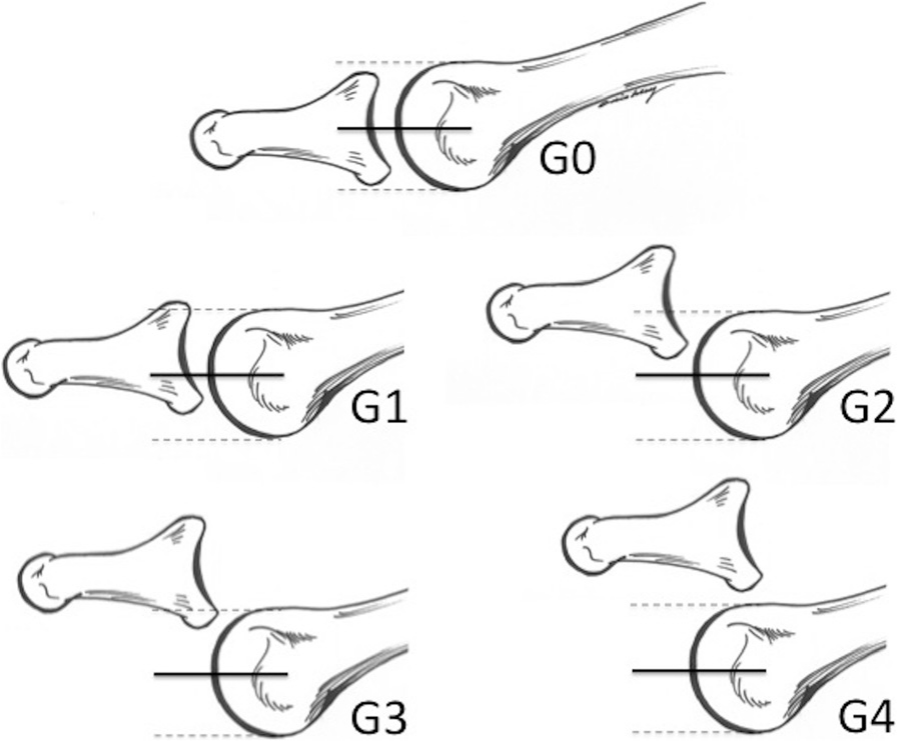
The graduation of Hamilton–Thompson MTP “drawer test”: G0 = stable joint, G1 = light instability (<50 % subluxable), G2 = moderate instability (>50 % subluxable), G3 = gross instability (dislocatable joint), G4 = dislocated joint

Joint instability was measured using the MTP “drawer” test [19] and rated as: G0 = stable joint, G1 = light instability (<50 % subluxable), G2 = moderate instability (>50 % subluxable), G3 = gross instability (displaceable joint), and G4 = dislocated joint (Fig. 2). Patients included in this study were those with grades 1 and 2 of joint instability measure by the drawer test. Patients with grade 3 or 4 were not included.

Pre- and post-operative weight-bearing radiographs in the anteroposterior (AP) and lateral view were obtained to measure the articular congruity, the distal metatarsal articular angle, and the metatarsal parabola. A pre-operative forefoot magnetic resonance imaging (MRI) (1.5T) was also obtained to identify possible associated injuries and improve the diagnostic accuracy.

**Fig. 3:**
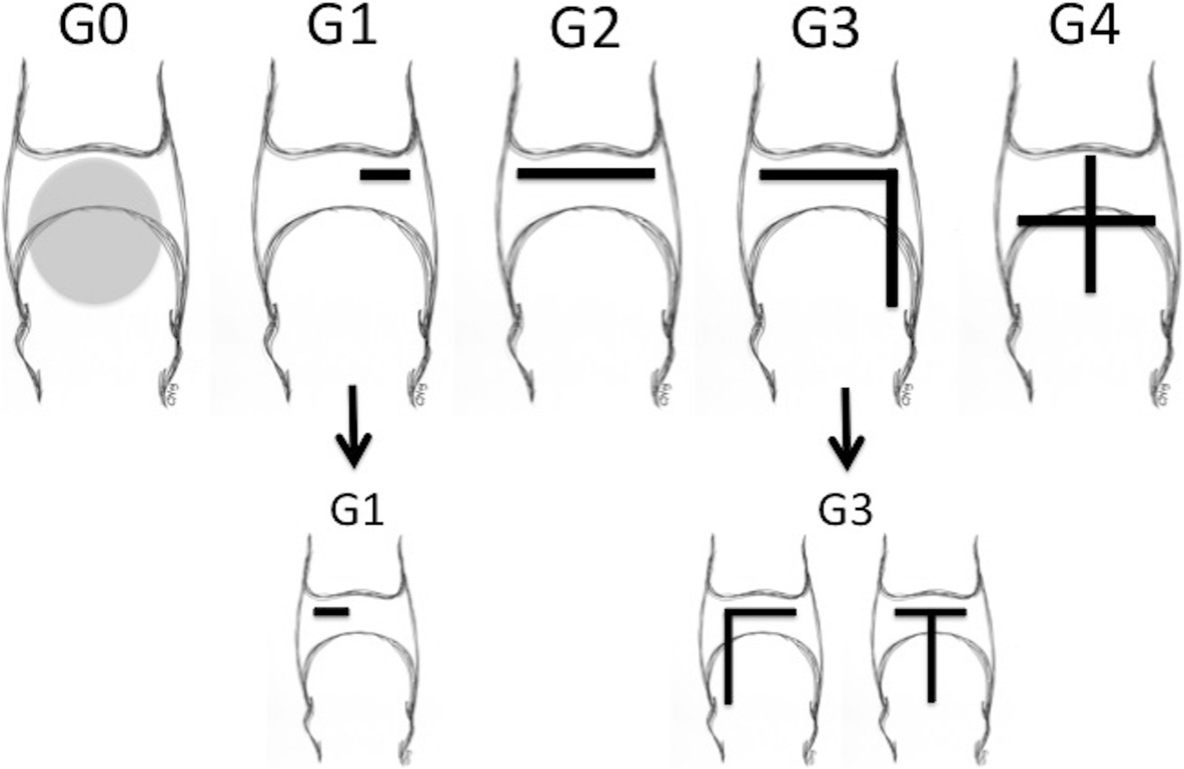
Schematic representation of the anatomic grading of MTP plantar plate lesions in a right MTP2 joint

After the clinical assessment, a diagnostic arthroscopy was performed to grade the PP tear (Fig. 3), confirming our physical examination. The diagnostic procedures were followed by the arthroscopic treatment steps used in the radiofrequency shrinkage of the plantar plate and the accessory collateral ligaments. After this, a small dorsal incision was used to perform the distal Weil metatarsal osteotomy.

Each patient was evaluated post-operatively every 6 months. The pre-operative and the last post-operative data were considered for statistical analysis.

### Surgical technique

**Fig. 4:**
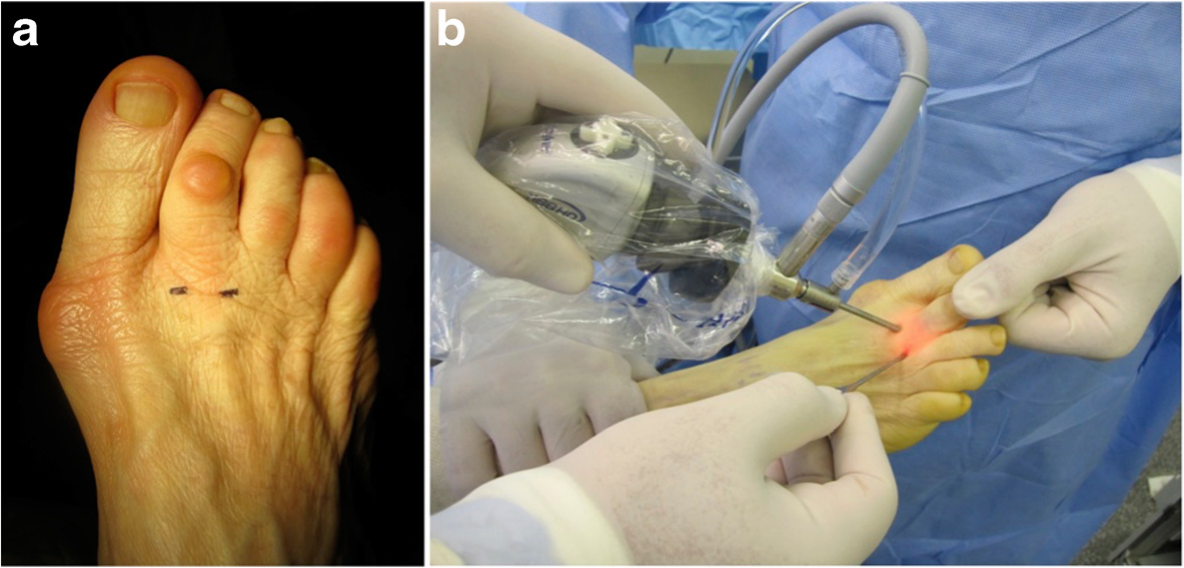
Arthroscopic settings. **a** The two dorsal MTP portals, dorso-medial, and dorso-lateral. **b** Positioning of the surgeon, facing the dorsal aspect of the foot

**Fig. 5:**
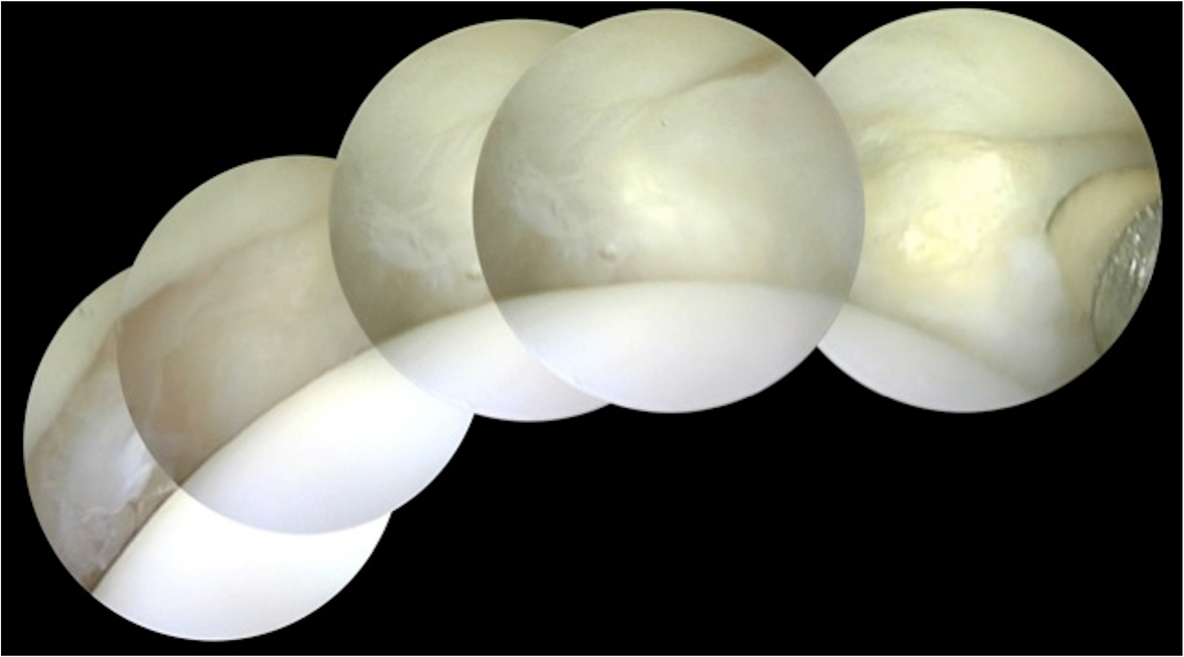
Panoramic view of a lesser MTP joint

**Fig. 6:**
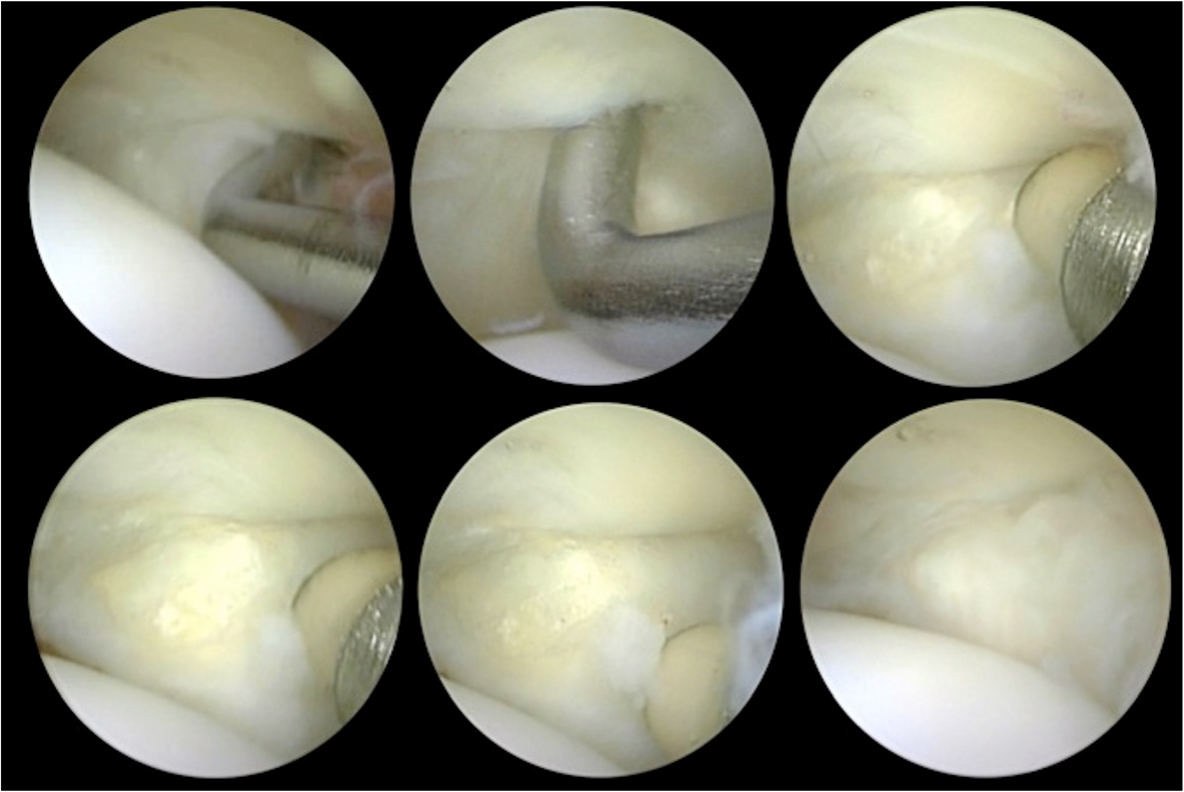
Multiple views of lesser MTP joint. Note that the arthroscopic probe tip is inside a small grade 1 plantar plate tear. The shrinkage and sealing of the lesion was obtained using radiofrequency as proposed in this study

All patients were treated by the same surgeon (C.N.). With a patient under regional block and sedation in a supine position, a tourniquet was applied at shin or thigh level and inflated at 300 mmHg after exsanguination and a 20-mmHg arthroscopic pump was used for irrigation. An arthroscopic evaluation of the involved lesser MTP joint was performed through two dorsal portals (medial and lateral portals placed over the MTP articular space) with a 2.7-mm, 30-degrees arthroscope (Fig. 4). Light manual traction was applied to the toe so that the central and distal portions of the plantar plate could be visualized, inspected, and then palpated with a probe. We performed a synovectomy of the affected joint and the PP lesions grades 0 and I were treated with radiofrequency (ArthroCare^®^ Short Bevel 25° 2.3 mm, Andover, MA, USA) shrinkage. The unit was automatically set to deliver a temperature of 60 °C (Figs. 5 and 6).

After the arthroscopic treatment of the PP, a Weil osteotomy through a dorsal approach using a sagittal saw was performed. The Weil osteotomy was then fixed in the desired position with one small snap-off (Spin^®^ Screw, Integra, Plainsboro, NJ, USA) self-tapping screw. After routine wound closure, a post-operative compression dressing was applied and the affected toes were held in 20° of plantar flexion at the MTP joint.

### Postoperative management

Following surgery, the stitches were removed at 3 weeks and the patients were allowed to ambulate in a forefoot non-weight-bearing shoe for 6 weeks, with the toe still being held in plantar flexion. Dressings were discontinued and comfortable shoes were permitted after 6 weeks following surgery. An exercise program was then initiated in order to condition the extrinsic and intrinsic muscles of the lesser toes. High-heeled shoes were prohibited until the sixth month post-operation.

### Statistical analysis

Variables analyzed in this study were summarized according to the groups (grade of pathology). In order to compare two independent groups the Student *t* test was used. The non-parametric Mann–Whitney *U* test was applied to compare three or more independent groups. The following statistical tests, complemented by multiple comparisons tests were used if a statistically significant *p* value was found:

- Analysis of Variance (ANOVA) with the fixed factor group complemented by the multiple comparisons tests of Bonferroni.
- Non-parametric Kruskal–Wallis test complemented by the multiple comparisons of Dunn’s tests.

The associations between the study groups and categorical variables were analyzed using the Pearson chi-square test or likelihood ratio chi-square test.

Variations between the results of the categorical variables in the pre- and post-surgery assessments were evaluated by McNemar’s test of symmetry.

Changes in post-surgery results in relation to the pre-operative evaluation of the numerical variables were evaluated by paired *t* test or non-parametric Wilcoxon signed-rank test.

The limit for rejection of the null hypothesis was considered to be 5 %.

## Results

Seventy-three percent of our patients were females with a mean age of 59 years. Most of them had a history of wearing high-heeled shoes (63 %) and only some reported sport activities (26 %). Bilateral involvement was more common than single appearance and our average follow-up was 20 months ranging from 12 to 47 months.

Table 1 reports the data regarding the number of affected joints. Sixty-five percent were grade 0 of the anatomical classification of plantar plate tear with a greater predilection for the third metatarsophalangeal joint. However, among the joints that were classified as grade 1, 58 % affected the second MTP joint. Sixty percent of the joints were considered acutely painful and 63 % demonstrated increase of the interdigital space.

**Table 1:** Demographic data of symptoms and affected joints

Data		Grade of lesion		Total
		0	1	
# Joints		23	12	35
Toe	II	5	7	12
		(21.7 %)	(58.3 %)	(34.2 %)
	III	17	3	20
		(73.9 %)	(25.0 %)	(57.1 %)
	IV	1	2	3
		(4.3 %)	(16.7 %)	(8.7 %)
Acute pain *n* (%)	No	10	4	14
		(43.5 %)	(33.3 %)	(40.0 %)
	Yes	13	8	21
		(56.5 %)	(66.7 %)	(60.0 %)
Swelling *n* (%)	No	18	5	23
		(78.3 %)	(41.7 %)	(65.7 %)
	Yes	5	7	12
		(21.7 %)	(58.3 %)	(34.3 %)
Spread toe *n* (%)	No	8	5	13
		(34.8 %)	(41.7 %)	(37.1 %)
	Yes	15	7	22
		(65.2 %)	(58.3 %)	(62.9 %)

Table 2 demonstrates the statistically significant difference between pre- and post-operative AOFAS scores (*p* < 0.0001), with an average increase of 34.5 points in the total sample. There was no statistically significant difference between the grades 0 and 1 in the AOFAS score average changes (*p* = 0.9842). For the VAS score, a statistically significant difference was observed between pre- and post-operative analysis (*p* < 0.0001), with a mean reduction of 6.9 in the total sample. Post-operative analyses of the VAS resulted in a statistically significant difference between grades 0 and 1 (*p* = 0.0340).

**Table 2:** Pre- and post-operative analysis of AOFAS score and VAS scale

AOFAS		Grade of lesion		Total
		0 (*n* = 23)	1 (*n* = 12)	(*n* = 35)
Pre-operative *n* (%)	Mean	56.2^#^	55.6^#^	56.0*
	Min–max	47–65	47–67	47–67
Post-operative (%)	Mean	90.7^#^	90.1^#^	90.5*
	Min–max	69–97	75–100	69–100
VAS				
Pre-operative *n* (%)	Mean	8.0&	7.3&	7.8*
	Min–max	6–9	7–9	6–9
Post-operative *n* (%)	Mean	0.91&	0.67&	0.83*
	Min–max	0–5	0–2	0–5

*p value (pre × post) *p* < 0.0001

^#^ *p* value [mean (G0 × G1)] *p* = 0.9842

&*p* value [mean (G0 × G1)] *p* = 0.0340

Table 3 presents the analysis of toe elevation and toe purchase. The grade 0 lesion showed a positive toe purchase, significantly higher than grade 1. It is interesting to demonstrate that 80 % of our patients had a positive toe purchase after surgery and more than 94 % of them had no toe elevation post-operatively. The individual analysis of the grades 0 and 1 regarding elevation showed a statistically significant result between the pre- and post-operative period with *p* values <0.0001 and <0.0016, respectively. However, no statistically significant difference between the groups was observed for the presence of elevation in the final evaluation (*p* = 0.1109). The toe purchase result was statistically significant. The grade 0 group showed more joints with toe purchase positive than grade 1 group. The individual and comparative analysis of the groups presented a statistically significantly different data (Table 3).

**Table 3:** Pre- and post-operative analysis of elevation and toe purchase

Elevation		Grade of lesion		Total
		0 (*n* = 23)	1 (*n* = 12)	(*n* = 35)
Pre-operative *n* (%)	No	5&	0^	5
		(21.7 %)	(0 %)	(14.3 %)
	Yes	18&	12^	30
		(78.3 %)	(100 %)	(85.7 %)
Post-operative *n* (%)	No	23*^,^&	10*^,^^	33
		(100 %)	(83.3 %)	(94.3 %)
	Yes	0*^,^&	2*^,^^	2
		(0 %)	(16.7 %)	(5.7 %)
Toe purchase				
Pre-operative *n* (%)	Present	2	2	4
		(8.7 %)	(16.7 %)	(11.4 %)
	Diminish	11	0	11
		(47.8 %)	(0 %)	(31.4 %)
	Absent	10	10	20
		(43.5 %)	(83.3 %)	(57.1 %)
Post-operative *n* (%)	Present	21^#^	7^#^	28
		(91.3 %)	(58.3 %)	(80.0 %)
	Diminish	0^#^	2^#^	2
		(0 %)	(16.7 %)	(5.7 %)
	Absent	2^#^	3^#^	5
		(8.7 %)	(25.0 %)	(14.3 %)

*p value (G0 × G1) *p* = 0.1109

^#^ *p* value (G0 × G1) *p* = 0.0340

&*p* value [(pre × post) grade 0] *p* < 0.0001

^^^ *p* value [(pre × post) grade 1] *p* < 0.0016

Table 4 demonstrates an important finding regarding the stability graduation. In the pre-operative period, 97 % of joints were classified as grade 1 of drawer test instability (<50 % subluxable). However, in the post-operative evaluation, more than 82 % of the joints were completely stable (grade 0 of instability). The individual improvement of the stability was statistically significant. In grade 0, 87 % of the joints became stable in the post-operative analysis (*p* = 0.0001) and in grade 1, 83 % of the joints improved in the pre-operative level of instability (*p* = 0.0139). The comparative analysis between the groups was not statistically significant.

**Table 4:** Pre- and post-operative analysis of stability and congruency

Stability		Grade of lesion		Total
		0 (*n* = 23)	1 (*n* = 12)	(*n* = 35)
Pre-operative *n* (%)	G0	0	0	0
		(0 %)	(0 %)	(0 %)
	G1	23	11	34
		(100 %)	(91.7 %)	(97.1 %)
	G2	0	1	1
		(0 %)	(8.3 %)	(2.9 %)
Post-operative *n* (%)	G0	20	9	29
		(87.0 %)	(75.0 %)	(82.9 %)
	G1	3	3	6
		(13.0 %)	(25.0 %)	(17.1 %)
	G2	0	0	0
		(0 %)	(0 %)	(0 %)
*p* value (G0 × G1)		0.3910		
Congruency				
Pre-operative *n* (%)	Congruent	18	7	25
		(78.3 %)	(58.3 %)	(71.4 %)
	Incongruent	5	5	10
		(21.7 %)	(41.7 %)	(28.6 %)
Post-operative *n* (%)	Congruent	23	11	34
		(100 %)	(91.7 %)	(97.1 %)
	Incongruent	0	1	1
		(0 %)	(8.3 %)	(2.9 %)
*p* value (G0 × G1)		0.3430		

Stability *p* value (pre × postoperative grade 0) *p* = 0.0001

Stability *p* value (pre × postoperative grade 1) *p* = 0.0139

Congruency *p* value (pre × postoperative grade 0) *p* = 0.0253

Congruency *p* value (pre × postoperative grade 1) *p* = 0.0455

Most of the joints were congruent in the pre-operative radiographic measurements (71 %) and we correlate these data to less extensive tears that our patients presented. The post-operative analysis demonstrates that less than 3 % of the joints had incongruent radiographic relations. The individual analysis of the groups was statistically significant (grade 0, *p* = 0.0253; grade 1, *p* = 0.0455) and the comparative analyses were not (*p* = 0.3430).

## Discussion

Currently, it is known that the plantar plate plays an important role in the stabilization of the lesser metatarsophalangeal joints. Recent biomechanical studies have shown that PP was the main isolated stabilizer of the MTP joints in the dorsal–plantar direction [4]. Deland and Sung [20] showed how an isolated repair of the collateral ligaments was insufficient to resolve instability resulting from PP deformity, which may indicate that the PP itself has a crucial role in stabilizing the joint.

In recent cadaveric dissections of crossover second toe deformities, using the anatomic grading system, progressive anatomic changes from lateral to medial were found, and the authors believed that this classification could assist pre-operative planning and performance of a surgical repair [21].

Multiple methods for correction of gross MTP joint instability have been described. The Weil osteotomy technique and the flexor-to-extensor tendon transfer are the most common [10, 22, 23]. Only recently, surgeons have advocated correcting the MTP joint instability by direct repair of the PP [2, 24–26]. Few of these reports use some type of classification to stratify the grade of MTP instability. The Weil osteotomy technique, on its own, has been one of the mainstays of surgical treatment for lesser MTP instability. In a prospective study with 7 years of follow-up, Hofstaetter et al. found 88 % patient satisfaction rate [23]. However, this author demonstrated 12 % of re-dislocation and 68 % incidence of floating toe. In a recent literature review, Highlander et al. [27] indicated that the floating toe was the most common complication of the Weil procedure, with a reported incidence average of 36 %.

The other current option in the literature is the flexor-to-extensor transfer. Gazdag and Cracchiolo [28] reported 35 % of fair results in a series of 20 feet, which were submitted to a flexor-to-extensor transfer. The relative success was attributed to post-operative stiffness. Myerson and Jung [29] reported on their retrospective group of 64 feet that underwent the same procedure and demonstrated that 34 % of patients had either major reservations or were unhappy with the outcome. Although several studies have confirmed the effectiveness of a flexor-to-extensor transfer, these investigations report variable levels of patient satisfaction, with results ranging from 51 % to 89 %. Also, incomplete correction after tendon transfer in patients with subluxated MTP joints has also been reported in the literature [30].

Direct repair of the plantar plate has gained recent attention, and this tendency is driven by the relatively poor results obtained in the treatment of these deformities, most of the time followed by high rates of failure and recurrence. A prospective study showing the results of direct open PP repair (grades 2 and 3 of the anatomical classification) presented a post-operative AOFAS forefoot score of 92 points on average and more than 68 % of the stable joint [8]. There was no joint instability recurrence reported.

Until now, we have only found reports that address how to treat the extensive plantar plate and collateral ligament tears with gross instability and obvious clinical presentation. The early stages of the pathological process, involving less extensive lesions and a subtle instability, have no current treatment options suggested in the literature. A delayed evaluation and treatment of plantar plate tears is quite common due to the lack of clear diagnosis in the early phases. Conservative treatment is frequently instituted and is effective in reducing the inflammatory period, but is not able to prevent both the progressive insufficiency and the failure of the plantar plate as a joint stabilizer [6, 11].

To the best of our knowledge, this series is the first to present a treatment option for subtle MTP joint instability. In pre-operative analyses, none of our patients had a completely stable joint and more than 97 % had grade 1 instability. With the combination of radiofrequency and Weil osteotomy, 83 % of the MTP joints were totally stable (grade 0) with more than 97 % congruent joints. We observed that 80 % of patients had a positive toe purchase test post-operatively and 94 % of them presented with toe touching of the ground with no residual elevation during our post-operative observation. The results obtained in grades 0 and 1 of PP tears were superior to those reported in grades 2 and 3 previously. This may indicate that treating MTP joint instability in the early stages could produce a better prognosis.

Although radiofrequency has been used in a large number of patients and different joints, this treatment is still controversial. On the basis of short-term clinical follow-up, the results of thermal capsulorrhaphy have shown both supportive and non-supportive outcomes [16, 31]. Thermal capsular shrinkage has become a common procedure for a variety of joint conditions usually associated with instability [12]. Some histologic studies demonstrate evidence of tissue repair and remodeling, rather than degeneration and necrosis, during a period of 3.5–62 months after thermal capsulorrhaphy [16, 32]. Success rates of thermal capsulorrhaphy seem to vary according to patient populations, thermal techniques, adjuvant procedures, and rehabilitation protocols. Electrothermal collagen shrinkage (ECS) in larger joints such as the shoulder and knee has resulted in disappointment [31]. In contrast, ECS has been used successfully in hand surgery and ankle lateral ligament repair [16, 33]. Some authors have clarified that thermal contraction of the anterolateral capsular-ligamentous structures of the ankle is quite similar to an anatomic repair type procedure [12, 15]. The molecular effects of RF energy on soft tissue have been described in numerous reports [15, 17, 33, 34]. One of the most important effects of RF energy is its great ability to shorten collagenous structures; hence, thermal capsulorrhaphy has been shown to produce an effective reduction in capsular volume and joint translation. Due to the biomechanical weakness of the thermally treated capsule, some authors have proposed post-operative immobilization to avoid the thermally altered joint capsule stretching before the completion of the biological repair process [12, 15, 34, 35]. For this reason, we decided to maintain our patients with the affected toes in plantar flexion for 6 weeks.

The limitations of our study could be the small number of patients analyzed, the absence of a control group, and the minimum follow-up period of 12 months. In our study, we described a new treatment for mild plantar plate tears with subtle instability, and during our follow-up, there was no progression or recurrence of the instability. Based on these findings, one could infer that diagnosing and treating instability of the lesser MTP joints in the early stages could prevent progression of the disease.

## Conclusion

The combination of radiofrequency shrinkage of the plantar plate and distal Weil metatarsal osteotomy is a viable option in the treatment of the early stages (grades 0 and 1) of lesser metatarsophalangeal joint instability.
